# Hyaluronic Acid-Based Biomaterials for Soft Tissue Repair and Wound Healing: Clinical Evidence and Emerging Applications

**DOI:** 10.3390/gels12070655

**Published:** 2026-07-22

**Authors:** Bogdan Mircea Măciuceanu Zărnescu, Diana Cristina Pîrvulescu (Bunea), Adelina-Gabriela Niculescu, Alexandru Scafa Udriște, Alexandru Mihai Grumezescu, Sebastian Vâlcea

**Affiliations:** 1 University of Medicine and Pharmacy “Carol Davila”, 8 Eroii Sanitari, 050474 Bucharest, Romania; bmaciuceanu@gmail.com (B.M.M.Z.); alexandru.scafa@umfcd.ro (A.S.U.); sebastian.valcea@gmail.com (S.V.); 2 Faculty of Chemical Engineering and Biotechnologies, National University of Science and Technology Politehnica Bucharest, Gh. Polizu St. 1–7, 060042 Bucharest, Romania; diana.pirvulescu@stud.fim.upb.ro (D.C.P.); agrumezescu@upb.ro (A.M.G.); 3 Research Institute of the University of Bucharest—ICUB, University of Bucharest, 050657 Bucharest, Romania

**Keywords:** hyaluronic acid, HA matrices, wound healing, regenerative medicine, hydrogels, tissue engineering, chronic wounds

## Abstract

Hyaluronic acid (HA) is a glycosaminoglycan that is found within the body and has both structural and signaling functions in the extracellular matrix. HA is biocompatible and biodegradable; it has a high water content and binds directly to certain cell-surface proteins. Due to these characteristics, it is considered a promising component for the design of biomaterials for regenerative wound healing. This review covers the most recent findings on the use of HA-based biomaterials in soft tissue repair, while also incorporating earlier, foundational studies relevant to the field, focusing on HA’s characteristics, cellular interactions, design, and preclinical and clinical results. The physicochemical characteristics of HA and their influence on cellular responses and tissue regeneration are discussed to show how material properties can be adjusted for specific therapeutic purposes. There have been great advances in chemically modified composite scaffolds and HA matrices, which offer better mechanical stability and controlled degradation. At the same time, new delivery systems have been built using HA, from nanoparticles to gene delivery platforms and growth factors, and these have given the material an active role as a therapeutic agent rather than just a passive one. This narrative review covers the clinical evidence for the effectiveness of commercial products for acute and diabetic wounds, as well as burns and chronic wounds, and discusses where their use is indicated. In the end, the current limitations of the research and future applications and directions are discussed.

## 1. Introduction

**Figure 1:**
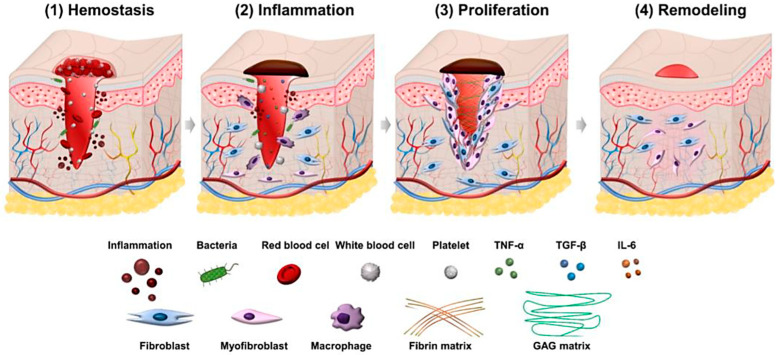
Wound healing phases. Reprinted from an open-access source [7].

Wounds are a major and growing clinical problem worldwide because they affect patients’ quality of life and can lead to other health risks. Chronic wounds alone are estimated to impact over 40 million individuals globally, with associated costs exceeding billions annually, particularly due to prolonged treatment, recurrence, and complications such as infection and delayed healing. On a global scale, wounds have become an increasingly significant clinical issue, not only for the health risks they pose but also for the toll they take on a patient’s quality of life. It is estimated that more than 40 million people around the world are affected by chronic wounds, draining resources each year, mainly because the treatment plan is prolonged, wounds can be recurrent, and there are also complications such as infection and delayed healing [1,2]. These wounds can be diabetic foot ulcers, venous leg ulcers, and pressure injuries, which do not usually follow the normal phases of healing: hemostasis, inflammation, proliferation, and remodeling. The wound healing phases are illustrated in Figure 1. This results in persistent tissue damage and increased morbidity. With older age groups growing and more people facing conditions like diabetes, the challenge is intensifying [3,4]. Even though medicine has advanced, conventional wound treatments are limited in their ability to promote rapid, complete healing. Traditional methods, such as gauze dressings, bandages, and topical agents, provide only physical protection and exudate absorption, without altering the wound microenvironment. These treatments continue to have healing gaps, particularly around preventing infections, forming new blood vessels, and cellular migration at damaged sites. Moreover, chronic wounds usually suffer from poor vascularization, excessive inflammation, and disrupted extracellular matrix (ECM) repair, which cannot be corrected using the mentioned conventional therapies alone. Because of these limitations, there is a clinical need for more advanced therapies that can actively support tissue regeneration [1,5,6].

Among current approaches, biomaterials play an important role in supporting regenerative medicine. To replicate the natural ECM, researchers designed materials that provide structural support while delivering bioactive stimuli to influence cellular responses [8]. Natural and synthetic polymers have been investigated for their potential use in wound healing. Natural polymers, such as collagen, chitosan, and alginate, have multiple benefits. They are biocompatible, biodegradable, and bioactive. These properties allow them to interact effectively with the wound microenvironment. Despite these advantages, limitations remain. Some limitations include weak mechanical properties and variability in biological performance, which may arise from differences in raw material composition, extraction methods, and patient-specific responses to the biomaterial. As a result, researchers have turned toward developing hybrid and functionalized systems [9,10]. The relevance of biomaterial-assisted defect reconstruction is also reflected in orthopedic settings [11,12], soft tissue regeneration [13,14,15], craniofacial reconstruction [16,17], and tissue engineering applications [18,19,20].

Among different biomaterials, hyaluronic acid (HA) stands out due to its unique biological and physicochemical properties. Found widely in nature, this glycosaminoglycan appears commonly in connective tissue of the ECM, with skin being one main location where maintaining moisture and elasticity depends on its presence. What makes it especially relevant is participation across every stage of tissue repair: inflammation, cell migration, angiogenesis, and re-epithelialization. Its water-binding and formation of gel-like networks help maintain moist conditions around wounds, supporting effective recovery. Beyond its physical effects, its engagement with surface receptors, such as CD44, alters signaling pathways that control tissue repair mechanisms [21,22].

The molecular weight of HA shapes its behavior in biological systems: high- and low-molecular-weight fragments influence inflammation and tissue repair in different ways. Given HA’s dual role as both a physical and signaling molecule, its use in designing novel biomaterials becomes feasible. HA can be easily chemically modified, is biocompatible, and has low immunogenicity. As a result, many HA-based materials have been developed, including hydrogels, sponges, and nanocarriers [21].

HA-based biomaterials could provide both structural support and biological function for soft tissue healing. Their framework supports cell regrowth while altering the wound microenvironment and also stimulates angiogenesis. Evidence now links such biomaterials to better outcomes in chronic wounds that resist standard care. Due to advances in materials science, it has been possible to engineer HA-based biomaterials with multiple functions, such as drug delivery, antimicrobial properties, and the ability to respond to wound-specific stimuli [1].

Because of these advantages, HA-based biomaterials represent a promising direction in regenerative medicine. Even though studies in animals and humans have delivered positive results, hurdles remain regarding consistent quality and sustained performance in everyday medical settings. This narrative review aims to provide an overview of HA-based biomaterials for soft tissue repair and wound healing, with a focus on clinical evidence and applications. In this paper, the biological functions of HA, the design and properties of HA-based biomaterials, their clinical performance in different wound types, and the latest innovations will be discussed.

## 2. Literature Search Methodology

This narrative review was conducted through a comprehensive literature search of relevant studies on HA-based biomaterials for wound healing and soft tissue repair. A literature search was conducted primarily in English-language publications using the PubMed database, and additional relevant publications were identified through ScienceDirect, SpringerLink, MDPI, and manual screening of references from selected articles.

Although the main focus was on recent publications up to 2026, several earlier foundational studies were also included to provide the required scientific background and to trace the evolution of HA-based biomaterials for wound healing.

The search strategy included combinations of the following keywords: “hyaluronic acid”, “hyaluronan”, “wound healing”, “soft tissue repair”, “hydrogels”, “biomaterials”, “tissue engineering”, “drug delivery”, “composite scaffolds”, “chronic wounds”, “diabetic wounds”, and “regenerative medicine”.

Studies were included if they: (i) investigated HA or HA-based biomaterials in the context of wound healing or other regenerative medicine and related biomedical applications; (ii) evaluated the physicochemical, biological, preclinical, or clinical performance of HA-containing systems; (iii) examined biomaterials, bioactive compounds, or therapeutic agents used in combination with HA; or (iv) provided relevant information regarding HA biological functions, properties, safety considerations, limitations, or clinical applications.

Studies were excluded if they: (i) were not relevant to the scope of HA-based biomaterials or regenerative medicine; (ii) did not provide information relevant to the objectives of this review; (iii) were conference abstracts, editorials, or not available in English.

## 3. Biological Role of HA in Tissue Repair

### 3.1. Structure and Physicochemical Properties of HA

**Figure 2:**
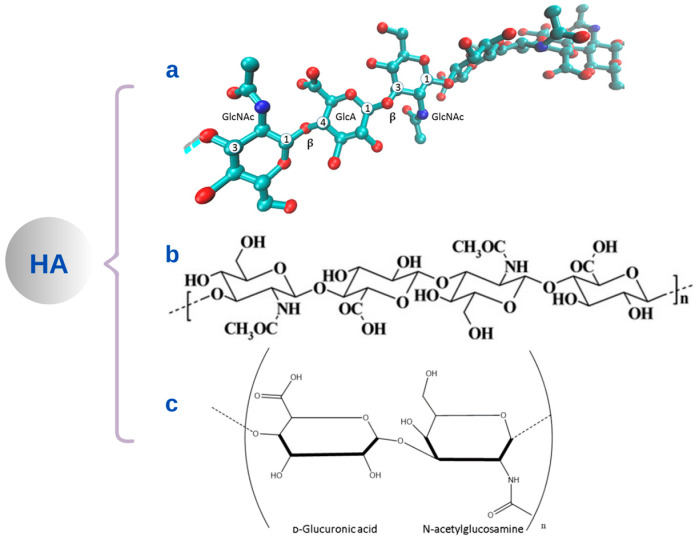
(**a**) 3D molecular structure of HA (GlcA-D-glucuronic acid, GlcNAc-N-acetyl-D-glucosamine); (**b**) chemical structure of HA; (**c**) chemical structure of an HA disaccharide. Created based on open-access sources [23,24,25].

HA is a linear, non-sulfated glycosaminoglycan composed of repeating disaccharide units of D-glucuronic acid and N-acetyl-D-glucosamine, which are linked alternately by β(1-3) and β(1-4) glycosidic bonds. Its chemical structure is illustrated in Figure 2 [23].

Unlike other glycosaminoglycans, HA is not sulfated and is synthesized directly at the plasma membrane by hyaluronan synthases, rather than in the Golgi apparatus. Its polyanionic structure, due to the presence of carboxyl groups, is responsible for its high electrostatic binding capacity for water molecules and cations, an important factor in its activity. This relatively simple structure of HA has made it a focus of chemical modification, making it a versatile material for biomaterial development, especially for hydrogels used in wound healing [21]. One of the most interesting properties of HA is its wide molecular weight (MW) distribution, ranging from small oligosaccharides to polymers exceeding several million Daltons. Furthermore, HA’s MW not only represents a physical characteristic but also determines how HA influences physiology and pathology. Take natural HA in normal tissues: it usually appears as a high-MW (HMW) polymer. It plays an important role in maintaining tissue homeostasis and protects against injury through its anti-inflammatory, immunosuppressive, and space-filling abilities. On the other hand, low-MW HA (LMW-HA) is produced following tissue injury. It acts as a signaling molecule that promotes the formation of new blood vessels and cell migration [26]. Experimental studies also show that the MW of HA has different effects on keratinocytes and fibroblasts. For example, HMW-HA is correlated with increased cell migration and proliferation, upregulation of growth factors, and influence on tissue repair [27].

The water-binding capacity is a defining trait of HA. Its molecules latch onto significant volumes of water relative to their own mass. This makes HA a hydrating component of the tissue, maintaining elasticity and a moist environment and enabling the transfer of nutrients through the ECM. Due to its strong water affinity and long polymeric structure, HA can form highly viscous, elastic networks. These are important in wound healing because HA-rich matrices offer mechanical support, protect against damage, and promote cell migration. It also contributes to the development of hydrated gels in the ECM. This helps to regulate the diffusion of cytokines, growth factors, and other metabolites and forms an ideal microenvironment for cellular processes (such as proliferation and differentiation) [21,28].

Inside the body, HA is found in several tissues, primarily in soft connective tissues such as skin, cartilage, synovial fluid, and vitreous humor. HA helps organize the ECM in the skin by maintaining dermal hydration equilibrium. Also, HA plays an important role in the provisional matrix that forms after tissue injury. Right after damage occurs, early repair frameworks depend heavily on its presence because it accumulates in the wound area. In this way, it creates a hydrated scaffold that facilitates cell migration and infiltration. As the healing process shifts through stages, both the quantity and MW of HA influence how HA supports rebuilding. HA also interacts with several cell-surface receptors, including CD44 and RHAMM, which mediate many of its biological effects during tissue repair. These receptor-mediated mechanisms are discussed in Section 3.2 [29,30].

In conclusion, HA has a simple structure, bioactivity based on its MW, the ability to bind water, and a wide distribution in connective tissues. These natural functions make HA vital to wound repair and make it a very useful tool for developing biomaterials to improve wound healing.

### 3.2. Cellular Interactions and Receptor-Mediated Signaling

Beyond supporting structure in the ECM, HA influences how cells act by attaching to particular receptors on their surfaces. These interactions are context-dependent and are crucial for coordinating the various phases of wound healing, including inflammation, proliferation, and tissue remodeling. The signals triggered by HA mainly come from interactions with surface proteins, especially CD44 and RHAMM. CD44 is a transmembrane glycoprotein found on many cell types, including fibroblasts, keratinocytes, and immune cells. When HA binds to CD44, it influences cell adhesion, migration, and proliferation and is an important mediator of intracellular signaling pathways, including MAPK and PI3K/Akt. Cell motility and cytoskeletal organization rely heavily on RHAMM. RHAMM is expressed on the cell surface but also intracellularly. It interacts with microtubules and intracellular signaling molecules, and these interactions lead to cell motility. Following tissue damage, broken-down HA activates signals through RHAMM, which guides fibroblasts and keratinocytes towards the wound site. The biological functions of HA during wound healing are influenced by the combined effects of its physicochemical properties and the cellular signaling mechanisms described previously [26,31].

HA can also influence inflammatory responses via TLR4. Specifically, when the enzymatic degradation that happens after injury generates LMW-HA, these fragments are recognized by TLR4. By activating TLR4-dependent signaling cascades, HA fragments promote the synthesis of inflammatory mediators that facilitate the recruitment of immune cells and early debridement of the wound site. So in addition to its structural role within the ECM, HA is also involved in the regulation of the inflammatory microenvironment. From a biomaterial point of view, HA degradation needs to be controlled because the presence of certain HA fragments can affect inflammation. Moreover, it was observed that TLR4 signaling has a dual effect on wound healing processes. Temporary activation of this pathway helps initiate the inflammatory response required for tissue repair, but prolonged activation can lead to persistent inflammation and delayed healing. Considering this, it is important to keep a balance between the pro-inflammatory effects of LMW-HA and the regenerative effects of HMW-HA [32,33,34,35].

In fibroblasts, HA–receptor interactions trigger signaling pathways involved in proliferation, migration, and matrix production. These promote fibroblast recruitment at the wound location and support ECM synthesis. In addition, these signals increase the expression of collagen types I and III. They also increase the expression of other ECM components, such as fibronectin, which fibroblasts then secrete outside the cell to help rebuild the damaged tissue [27,32]. Beyond promoting fibroblast growth and movement, HA influences their differentiation toward myofibroblasts, which is important for wound contraction during the healing process. The effects of HA on fibroblast differentiation into myofibroblasts are dependent on both the concentration of HA and the molecular size of HA. Specific HA fragments promote the expression of α-Smooth Muscle Actin (α-SMA), a marker of myofibroblast activation [36,37]. HA also controls how much matrix is kept or removed. Fibroblasts produce enzymes called matrix metalloproteinases (MMPs) that degrade the ECM, and inhibitors called TIMPs that block this degradation. HA signaling influences the expression of both. Through receptor-mediated signaling, HA can increase or decrease MMP and TIMP production depending on the healing stage, thereby ensuring that early matrix is not removed too quickly. It also ensures that it later gets remodeled into a more organized structure. This balance prevents either excessive scarring or incomplete repair [36].

In keratinocytes, HA contributes to re-epithelialization. This is done by supporting coordinated cell migration at the wound location. Also, the hydrated pericellular matrix formed by HA facilitates cell movement and helps maintain a microenvironment that is suitable for epidermal regeneration. HA also modulates the expression of adhesion molecules and genes associated with growth. Together, these support the restoration of epidermal integrity and barrier function [28,38].

The formation of new blood vessels, known as angiogenesis, supports recovery during wound repair, as developing tissue requires a constant supply of oxygen and nutrients. This process is primarily conducted by endothelial cells, which line the inside of blood vessels. HA influences how these endothelial cells behave, especially after it is broken down into smaller pieces in the wound. When the tissue is injured, enzymes break down large HA molecules into LMW fragments. These smaller fragments bind to specific receptors on endothelial cells (such as CD44 and RHAMM). Once binding to these receptors occurs, it triggers intracellular signaling pathways (ERK1/2). Then, these pathways transmit signals from the cell membrane into the nucleus, and here they activate genes involved in cell division and migration. This represents the main processes for neovascularization. HA plays another role in this process: it amplifies it by increasing production of VEGF and other angiogenic factors. HA continuously provides signals to endothelial cells, so they proliferate, migrate, and form new blood vessels. Several cytokines also signal cells to proliferate. Therefore, through the mechanisms described, HA promotes new blood vessel formation, which is very important for delivering oxygen and nutrients to the tissues during the healing process [27,39].

### 3.3. Role of HA in Wound Healing

Throughout tissue repair, HA participates in all stages of healing through the combined effects of its physicochemical properties and the receptor-mediated cellular interactions described above. A summary of HA functions across different wound healing phases is presented in Table 1.

In the early stages of inflammation, HA levels are rapidly elevated at the lesion site due to increased production by fibroblasts and keratinocytes. The presence of HMW-HA is important for maintaining tissue hydration and establishing a provisional matrix that allows immune cells to pass through. On the other hand, LMW-HA fragments act as danger-associated molecular patterns (DAMPs), initiating inflammation by activating receptors such as CD44 and TLRs [40]. Because of its ability to bind to water, HA creates moist surroundings that support the migration of fibroblasts and keratinocytes. Macrophages, when exposed to HA, often switch from aggressive, pro-inflammatory M1 forms toward healing M2 types. This prevents excessive tissue damage and prolonged inflammation. Later on, during the proliferative phase, HA is critical for cell migration and growth. HA forms a moist environment, which is essential for keratinocyte and fibroblast migrations. This process contributes to re-epithelialization and the development of granulation tissue. This creates a synergistic effect, which is very beneficial in regenerative medicine [27,41].

HA also influences angiogenesis through its interactions with endothelial cells. Instead of acting alone, it supports fresh vascular networks that deliver essential supplies during healing. Its pro-angiogenic activity contributes to the formation of new vascular networks that are needed for tissue regeneration and metabolic support during healing [39].

Later in healing, during remodeling, hyaluronidase breaks down HA, reducing its concentration over time. Because of this breakdown, collagen synthesis is positively affected, as is proper tissue formation. Collagen synthesis and alignment are affected by HA, as it influences matrix remodeling. This has effects on the nature of scars. Studies show that when HA remains elevated, fibrotic scarring tends to be less common [42].

## 4. HA-Based Biomaterials: Types and Design Strategies

### 4.1. HA Hydrogels

HA hydrogels are among the most widely researched biomaterials for wound healing. This is because of their natural properties, such as biocompatibility, high water content, biodegradability, and similarity to the natural ECM. Linking HA chains into networks creates three-dimensional structures capable of trapping moisture while maintaining structural integrity; this process is called crosslinking. HA hydrogels can be crosslinked physically (ionic interactions, hydrogen bonding, hydrophobic associations, guest–host interactions) or covalently, with chemical crosslinking usually having better mechanical stability and more predictable degradation [43,44].

Chemical crosslinking is the most common method. It changes the functional groups of HA, such as hydroxyl and carboxyl groups. Esterification, amidation, and click chemistry reactions are some of the chemical crosslinking methods. For instance, methacrylated HA (MeHA) can be photo-crosslinked with UV or visible light, which provides very precise control of the speed of gelation and the mechanical properties [45].

Unlike chemical methods, physical crosslinking occurs through forces such as hydrogen bonds, ionic interactions, and hydrophobic interactions. These hydrogels are generally reversible and injectable but tend to have lower mechanical strength and faster degradation rates [46].

Dynamic covalent crosslinking represents another type of crosslinking used. Unlike conventional chemical crosslinking, which generates permanent covalent networks, dynamic covalent bonds such as Schiff base (imine), hydrazone, oxime, and boronate ester linkages can be reversed and reform under physiological conditions. These characteristics enable the formation of self-healing, injectable, and stimuli-responsive hydrogel systems that are very useful for highly mobile and chronic wounds. One of the most common techniques to create HA-based hydrogels is the formation of the Schiff base reaction between aldehyde-modified HA and amino-containing polymers. This technique does not require any harsh initiators or reaction conditions [47,48]. Similarly, boronate ester bonds formed between boronic acid groups and cis-diol-containing molecules provide reversible crosslinking and responsiveness to changes in pH, glucose concentration, and oxidative stress, which could make them useful for diabetic wound treatment [49]. Compared to physically crosslinked hydrogels, dynamic covalent crosslinked hydrogels offer greater mechanical stability and structural integrity while retaining reversible properties. The process of physical crosslinking can be beneficial due to its simple synthesis process and mild fabrication conditions. However, it can result in limited mechanical strength, poor resistance to dilution, and rapid degradation in the case of very exudative wounds. Despite their benefits, the reversible nature of dynamic covalent bonds can lead to lower long-term mechanical stability and faster degradation than permanently crosslinked networks. Therefore, careful consideration is needed when choosing the crosslinking method, depending on the desired properties and wound type [46,48,50].

Injectable hydrogels made up of HA have gained attention because of their minimally invasive delivery. These injectable forms adapt easily to irregular wound shapes. By using specific stimuli, like changes in temperature or pH, or enzyme action, these hydrogels can be triggered to form gels.

Thermosensitive HA-based hydrogels, which are often mixed with polymers like chitosan, stay liquid at room temperature and turn into a gel when they are exposed to physiological temperature. This method makes the application easier and supports long-lasting retention at the wound [51].

Adding bioactive substances or even combining HA hydrogels with other biomaterials could increase their efficiency in wound healing. By doing this, researchers could address limitations such as the hydrogel’s low mechanical strength and its lack of biological activity. For example, growth factors such as VEGF and EGF could be incorporated into the hydrogel’s composition, enabling their controlled delivery to the wound site and promoting angiogenesis and overall healing [52]. On the other hand, the introduction of antibacterial substances, such as silver nanoparticles (Ag NPs) or antibiotics, can prevent chronic wound infections [53].

### 4.2. Chemically Modified HA Matrices

HA is very well tolerated due to its biocompatibility and natural presence within the body. However, it has some limitations: it is rapidly degraded by enzymes and has poor mechanical strength, which poses challenges for its use as a scaffold in soft tissue engineering. To overcome these drawbacks, multiple studies have been conducted on chemically modified HA-based scaffolds [54].

The chemical modifications can be done through techniques that involve the hydroxyl, carboxyl, or N-acetyl functional groups present in the structure of HA. This makes it easier to incorporate reactive functional groups that can make crosslinking or bioconjugations possible. Common modification strategies include esterification, amidation, thiolation, and methacrylation, each designed to improve the rapid degradation and limited mechanical strength of native HA while preserving its excellent biocompatibility [43].

Among these approaches, esterification and amidation are the most widely used because they enable significant modification of HA’s properties without compromising its biocompatibility. Esterification involves the reaction of HA carboxyl groups with alcohols, yielding derivatives with reduced hydrophilicity and slower degradation rates. In contrast, amidation forms amide bonds between HA carboxyl groups and amine-containing molecules, facilitating the conjugation of bioactive compounds, peptides, proteins, or synthetic polymers. Thiolation introduces sulfhydryl groups into the HA backbone, allowing the formation of disulfide bonds under physiological conditions and thereby enabling the fabrication of injectable, self-healing hydrogels [55,56,57].

Most research on HA derivatives for wounds focuses on MeHA. It is made by reacting HA with methacrylic anhydride or glycidyl methacrylate, adding methacrylate groups that can be polymerized under UV light [58,59]. The degree of methacrylation can be adjusted to control crosslink density, swelling, degradation rate, and stiffness. MeHA hydrogels have shown promising results in preclinical wound models, including faster wound closure, improved neovascularization, and reduced scar formation [59].

Another class of chemically modified HA matrices is represented by HYAFF technologies, which involve benzyl ester derivatives of HA. Unlike MeHA, which is primarily developed for hydrogel fabrication, HYAFF materials are designed to enhance HA stability while maintaining its biological activity. HYAFFs have much lower hydrophilicity and slower degradation rates than natural HA and can be produced in solid forms, such as films, meshes, sponges, and nonwoven tissues [56,60]. The extent of esterification affects the properties: semi-esterified forms, such as HYAFF-11 (around 75% esterification), retain some hydration while maintaining sufficient mechanical strength for manipulation [56].

### 4.3. Composite HA Scaffolds

Although HA is biocompatible and bioactive, it faces challenges due to its rapid degradation, poor mechanical strength, and inadequate cell adhesion properties, making it difficult to use independently as a tissue engineering scaffold. This is why composite scaffolds containing HA, along with other polymer materials, have been developed [61,62].

Collagen–HA composites are among the most promising approaches for regenerating soft tissues, as they mimic key components of the skin’s ECM. The function of collagen is to provide mechanical strength and attachment points for cells, while HA provides hydration, viscoelasticity, and signal-transmission capacity. Together, they create a support system for promoting fibroblast proliferation and angiogenesis [63]. One experiment found improved healing in guinea pigs using a collagen–HA matrix created by EDC crosslinking, compared with untreated wound models. Among the investigated formulations, the scaffold containing 9.6% HA showed the highest in vitro fibroblast proliferation. In vivo, collagen–HA matrices led to thicker dermal tissue formation, accelerated epithelial regeneration, and increased collagen synthesis compared with untreated controls. However, when the authors increased HA content, no significant reduction in wound size was observed. This suggests that its primary contribution was to tissue quality and matrix remodeling [64]. Ying et al. developed an in situ-forming collagen–HA hydrogel, which promoted wound healing and collagen deposition in diabetic wounds. They used a full-thickness wound model. A wound closure rate of 96.44 ± 0.47% was observed after 14 days, which was higher than those of the commercial treatment (85.97 ± 1.51%) and the individual component hydrogels (~93.8%). Also, the hydrogel produced the thickest granulation tissue (~1300 μm), confirming improved tissue regeneration [65].

Additionally, collagen–HA matrices are less prone to degradation and can maintain their structure throughout the healing process. This is important in an environment containing proteases, because the collagen scaffold alone would be degraded prematurely. Moreover, the addition of HA improves the matrix’s ability to retain moisture and nutrients [66].

HA–chitosan scaffolds represent a powerful combination, as they benefit from the synergistic effects of both polysaccharides: chitosan has antimicrobial activity, supports coagulation, and carries a positive charge, while HA is responsible for hydration, signaling via CD44 receptors, and anti-inflammatory activity. Their opposing charges, the negative charge of HA and the positive charge of chitosan, aid in the formation of the polyelectrolyte complex. This complex can be modified in terms of porosity, swelling properties, and degradation rate [67]. These HA–chitosan composites have shown improved wound healing performance due to several factors: modulation of inflammation, promotion of granulation tissue formation, and antibacterial effects. In a study by Deng et al., an HA–chitosan hydrogel improved tissue regeneration by promoting angiogenesis, ECM deposition, and macrophage polarization toward the pro-healing M2 phenotype. In vivo, it produced a 2.5-fold increase in regenerative tissue thickness compared with controls. It also demonstrated increased collagen deposition and higher VEGF and TGF-β expression [68]. Similarly, gelatin–HA composite hydrogels have been shown to accelerate re-epithelialization and improve tissue organization. The hydrogels provided a moist wound environment, which is important for tissue repair. In vitro experiments demonstrated good cytocompatibility and cell proliferation, especially for the gelatin–HA 8:2 formulation. In vivo, all hydrogel-treated groups experienced accelerated wound healing compared with controls, and the best result was achieved with the gelatin/HA 8:2 hydrogel [69].

HA can also be modified with synthetic polymers such as poly(ethylene glycol) (PEG), polycaprolactone (PCL), and poly(vinyl alcohol) (PVA). This type of combination offers benefits such as greater flexibility and tensile strength, making the biomaterial more suitable for applications that require weight-bearing capabilities and durability [70].

Synthetic polymers can slow degradation, prolonging the scaffold’s lifespan throughout all stages of the healing process. The addition of HA to synthetic polymers improves biocompatibility and reduces the risk of toxicity that can occur when using synthetic polymers alone [71]. Recent studies have demonstrated the importance of the interactions among scaffold composition, microstructure, and mechanical properties for therapeutic outcomes. More specifically, higher porosity and interconnected pore networks improve cell infiltration and vascularization. Stiffer parts guide stem cells toward differentiation and tissue remodeling. Composite hydroxyapatite–HA scaffolds represent a way to adjust these parameters, which supports the design of next-generation biomaterials [72]. Examples of composite HA scaffolds, their properties, and biological effects are presented in Table 2.

The studies summarized in Table 2 demonstrate that the performance of HA-based composite scaffolds is influenced by their physicochemical and mechanical properties. Porous structures with pore sizes ranging from a few micrometers to over 100 μm facilitate nutrient diffusion and cell infiltration. The high porosity allows the transportation of oxygen and manages the exudate. The mechanical properties are also important. In these publications, the tensile strength varies from 0.63 to 1.12 MPa, and compressive strength reaches 35.2 N·mm. This provides the necessary stiffness of wound dressings, and it also keeps them flexible [75,76,78]. Researchers reported strategies to increase scaffold stability using polydopamine coating or polymer crosslinking. These scaffolds showed improvements in degradation compared to HA-based scaffolds without additional modifications [73,78]. Such multifunctional characteristics support cell proliferation, angiogenesis, ECM remodeling, and wound closure simultaneously.

### 4.4. Advanced HA-Based Delivery Systems

The development of advanced HA delivery systems represents an improvement from simple passive biomaterials to more complex, active systems that can modulate the wound microenvironment. This can be achieved through the physical properties of chemically modified HA, together with its innate biological affinity for receptors on cell surfaces. Unlike their biological role, as discussed in Section 2, the physical properties of HA can also be used as targeting mechanisms that promote the adherence and retention of HA-based carriers by cells participating in wound healing. This targeted delivery can improve local drug accumulation, reduce off-target effects, and improve the therapeutic efficacy of incorporated bioactive agents [81,82].

**Figure 3:**
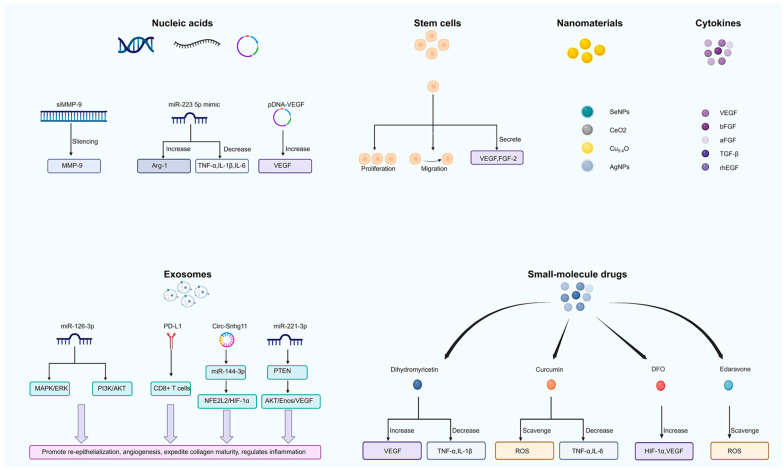
Examples of wound healing agents and molecules that can be used in drug delivery systems. Reprinted from an open-access source [83]. © 2023 Hao, Wang, Duan, Kan, Li, Wu, Xiang, and Liu.

HA hydrogel scaffolds can serve as drug delivery systems, with therapeutic agents incorporated into the polymer matrix. Examples of wound healing agents and molecules that can be combined with HA are illustrated in Figure 3.

Research shows that NPs combined with HA can reach deeper skin layers, helping treat burns and chronic wounds more effectively. For example, HA-functionalized chitosan NPs co-loaded with curcumin and quercetin penetrated deeper into the skin layers (2414 μg/cm^2^ for curcumin and 1984 μg/cm^2^ for quercetin), where they exhibited sustained drug release and prolonged retention within skin tissues. These led to accelerated tissue regeneration: ~98% wound closure by day 28 [84]. One of the functions of HA-based delivery systems is the ability to deliver the drug or substance in a controlled manner. This is done by chemically modifying HA to form hydrogels, nanogels, or other hybrid matrices that degrade in a controlled manner, thereby providing sustained drug release. Even more controlled release can be achieved using stimuli-responsive mechanisms, meaning the drug release can be influenced by environmental factors, such as pH, enzymes, or oxidation. Oxidation is especially beneficial for chronic wounds because their pathological environment differs from that of normal, healthy tissue [85,86]. A study has demonstrated that pH-responsive hydrogels, based on Schiff base crosslinking between oxidized HA and chitosan, degrade faster in an acidic environment (pH 5.5–6.5). This is similar to the pH in chronic wounds; therefore, it can provide controlled drug delivery to target areas [87,88]. In another recent study, four times faster degradation was noted at pH 5.5 than at pH 7.2. Additionally, under acidic wound conditions, metformin release promoted M1-to-M2 macrophage polarization, which in turn stimulated fibroblast migration and collagen production [87]. Hydrogels responsive to temperature changes and antimicrobial peptides are among other potential types that are being researched [88,89].

Growth factor delivery using HA-based systems also draws interest because wounds need these factors to heal properly. These growth factors are essential in processes such as angiogenesis, cell proliferation, and ECM formation. Despite these advantages, the clinical application of growth factors may be limited by their rapid degradation [52,90]. HA–chitosan NPs, for instance, have proven effective at delivering both VEGF and PDGF-BB. This improved blood vessel formation and tissue regeneration. The encapsulation was high (94% for VEGF and 54% for PDGF-BB), and the system was biocompatible, providing sustained PDGF-BB release for approximately 1 week. This observation could be further studied for potential use in promoting vascularization in regenerative applications [91]. According to Prestwich, crosslinked HA hydrogels may act as reservoirs of VEGF, PDGF, and BMP-2 for weeks or even months. This action preserves their bioactivity and enables prolonged therapeutic release [92].

Using HA-based drug carriers in regenerative medicine builds complex systems that provide structural support while releasing active substances to enhance healing.

Besides carrying standard drugs and growth factors, HA-based drug delivery systems are now also used to deliver living cells and genetic material to improve healing outcomes. Modern HA-based nanodelivery systems could be capable of recruiting stem cells. In this way, they induce anti-inflammatory responses and promote angiogenesis, both of which are beneficial for wound repair [93]. HA-based delivery systems can also be used for gene therapy. This includes using siRNA and miRNA therapies to target specific molecules that promote healing [94]. By combining targeted delivery, controlled release, and bioactive functionality, these systems address multiple aspects of the healing process simultaneously.

## 5. HA Biomaterials in Wound Healing: Evidence

### 5.1. Preclinical Evidence

Preclinical experiments provide the primary evidence for how HA-based biomaterials could promote wound healing. These experiments, ranging from in vitro assays to animal models, have demonstrated that HA-based materials can enhance cellular activity and promote wound closure and tissue regeneration. Various in vitro techniques (e.g., scratch assays) have been used to assess the effects of HA-based materials on cell migration and proliferation. Such in vitro assays reproduce key early wound healing events and enable the evaluation of cellular responses to HA-based biomaterials. In a study by Zhang et al., the researchers observed that HA-based systems show improved cell migration rates and faster wound closure. In this study, modified HA hydrogels promoted the proliferation of fibroblasts, stem cells, and endothelial cells, increased anti-inflammatory IL-10 production (57.9–68.1 pg/mL), and also induced early angiogenesis without causing chronic inflammation (after 28 days of implantation) [95]. For example, HA-based hydrogels combined with polynucleotides improved fibroblast migration and achieved near-complete closure within 24–48 h in scratch assays. The combination increased clonogenic efficiency approximately 3-fold compared with controls and promoted the formation of dense and multilayered colonies. The hydrogel also increased the expression of collagen types I and III [96]. In another study, alginate–HA composite dressings promoted early-stage wound closure and increased cellular activity compared with the control. Compared with alginate-only hydrogels, alginate–HA demonstrated better scratch closure in stem cells and keratinocytes (*p* < 0.01 and *p* < 0.001, respectively). This formulation sped up wound closure in a rat excisional wound model after 5 days (*p* < 0.001). These findings support the beneficial effect of HA on wound healing [97].

In vivo studies have also provided important evidence for these biomaterials. Research using murine full-thickness excisional wound models, which are common preclinical models, suggests that HA hydrogels and composite scaffolds support wound closure, granulation tissue formation, collagen synthesis, and neo-epithelialization [98,99]. For example, an HA-based glucose-responsive antioxidant hydrogel was able to improve wound closure and promote re-epithelialization in cases of diabetic wounds. The hydrogel released catechin in response to elevated glucose levels, and this reduced oxidative stress. In addition, it promoted angiogenesis by increasing VEGF and CD31 expression and suppressed inflammation by decreasing IL-6 and increasing IL-10 levels. These combined actions led to accelerated diabetic wound repair—within 3 weeks. This evidence is significant, since diabetic wounds can be very difficult to treat [100]. Trabucchi et al. demonstrated that LMW-HA protects granulation tissue against oxidative stress, demonstrating that it plays a cytoprotective role [99]. In another study, using rabbit full-thickness wound models, HA-based hydrogels promoted skin regeneration and improved the structural organization of the newly formed tissue. After 56 days, the HA hydrogel accelerated wound healing and reduced scar formation compared with HA powder and cotton-dressing controls. It increased VEGF and α-SMA expression, which supports angiogenesis and tissue regeneration. It also reduced TGF-β1 expression during the early healing phase, which reduced inflammation [101].

In diabetic wound models, which better mimic the impaired healing seen clinically, HA-based biomaterials also showed great results. HA hydrogels loaded with MSCs, adipose-derived stem cells, or growth factors have achieved almost complete wound closure in streptozotocin-induced diabetic rats, outperforming both unloaded HA scaffolds and conventional dressings [65,87]. In situ-forming collagen–HA hydrogels positively influenced collagen deposition, angiogenesis, and inflammation resolution in diabetic wounds. This showed that HA can target several pathological processes at once [65]. A summary of several preclinical studies is presented in Table 3.

The studies summarized in Table 3 demonstrate that HA-based biomaterials promote wound healing through multiple complementary mechanisms, which were also detailed in the previous sections. Most formulations improved wound healing rates and tissue regeneration compared with control groups. Advanced hydrogel formulations had even more beneficial effects, as they incorporated substances with unique properties. These had functions such as antioxidant activity, antimicrobial activity, stimulus responsiveness, or the ability to deliver therapeutic agents. Another important observation is that HA-based systems have been shown to offer benefits in more complex healing conditions, including diabetic wounds. In these conditions, the biomaterials improved wound closure, promoted vascularization, supported fibroblast activity and collagen deposition, and reduced prolonged inflammation, all of which are important factors associated with delayed healing.

All these findings support the positive effect of incorporating HA into wound healing treatments. However, direct comparisons between studies are limited by factors that can influence research results, such as experimental design, wound models, and reported quantitative parameters.

### 5.2. Clinical Evidence

Clinical evidence spans diverse wound types, formulations, and research designs. Clinical evidence comes from both controlled trials and real-world observational studies. One of the earlier and most comprehensive overviews of clinical research findings is the systematic review and meta-analysis by Voigt and Driver on randomized control trials (RCTs), which included ten RCTs and 599 patients. It involved the use of HA derivatives in burns, diabetic foot ulcers, venous leg ulcers, neuropathic ulcers, and surgical wounds. HA derivatives showed better wound healing outcomes than conventional treatments. For example, in diabetic foot ulcers, a 50% reduction in ulcer area was achieved in 40 days, compared with 50 days in the control group. In neuropathic ulcers, HA derivatives improved healing at 12 weeks (RR = 0.24, 95% CI 0.24–0.49). Although the wound types were different, the overall findings provided important real-world evidence that supports the therapeutic value of HA in wound healing [106]. More recently, in 2025, a systematic review and meta-analysis focused specifically on diabetic foot ulcers. The analysis included seven RCTs with a total of 444 patients and demonstrated that HA-based therapies increased the probability of complete ulcer healing (OR = 3.92, 95% CI 1.74–8.81) and reduced the time to wound closure compared with control treatments, without an increase in adverse events. These findings are consistent with the conclusions of the earlier meta-analysis and further confirm the beneficial effects of HA on chronic diabetic wounds [107].

The results obtained in these meta-analyses are supported by additional evidence from RCTs. For example, in patients with venous or mixed-origin leg ulcers, an HA-containing gauze pad demonstrated improved wound healing after 45 days compared with the control (73% vs. 46%, *p* = 0.011), followed by higher healing rates at both day 45 (31.1% vs. 9.3%) and day 60 (37.8% vs. 16.3%). Lower pain intensity during treatment was also reported [108]. These findings were later reinforced by a larger multicenter RCT involving 168 patients. In this case, complete ulcer healing was achieved in 39.8% of patients treated with the HA-impregnated gauze pad compared with 18.5% in the control group (*p* = 0.002) [109]. These consistencies in results across independent studies further confirm that HA-based dressings play a beneficial role in the healing of chronic wounds while maintaining a good safety profile. In a prospective randomized placebo-controlled study evaluating a pure HA dressing (Healoderm) in diabetic foot ulcers, patients benefited from a higher complete healing rate than those receiving conventional dressings (84.6% vs. 41.6%, *p* = 0.041). Moreover, the median time needed to achieve a 50% reduction in ulcer size was almost 2 times shorter (21 vs. 39 days, *p* = 0.0127). No additional bioactive agents were incorporated into this dressing, demonstrating the therapeutic effect of HA itself on diabetic wound healing [110].

Other studies also demonstrated that in the case of chronic ulcers, including venous and diabetic ulcers, HA-based products led to reduced wound size and faster healing. For example, clinical trials on HA-based products showed better wound closure rates and granulation tissue formation in chronic ulcers, even in difficult wounds [111]. Uccioli et al. conducted a multicenter RCT of two-step autologous grafting using HYAFF scaffolds in 180 patients with difficult diabetic foot ulcers, finding a faster 50% reduction in ulcer area and higher complete healing at 20 weeks in the treatment group [112].

Another important benefit of HA-based biomaterials used in wound healing is the modulation of inflammation and a reduction in infection risk. This can be achieved through different formulations and the incorporation of antibacterial agents into the HA material. Clinical studies have reported reduced signs of inflammation (such as erythema, edema, and exudate) in wounds treated with HA-based dressings [113]. HA combined with antimicrobial agents (such as silver) has demonstrated effective control of bacterial load in chronic wounds, leading to better healing [114]. Other key clinical studies and their outcomes are presented in Table 4.

As summarized in Table 4, clinical studies investigating HA-based treatments have demonstrated beneficial effects across multiple wound types, including burns, diabetic foot ulcers, radiation, and surgical wounds. However, their comparison remains limited by the heterogeneity in study design, patient populations, wound types, and outcome measures. The included studies range from RCTs to prospective and comparative observational studies.

A consistent finding across most studies is the acceleration of re-epithelialization and wound closure when using HA-based treatments. Clinical benefits were observed in both acute and chronic wounds, including shorter healing times, faster wound closure, improved scar appearance, reduced postoperative morbidity, fewer adverse effects, and greater patient satisfaction. Based on the provided studies, it is also observed that simpler formulations primarily provide hydration, maintain a favorable wound microenvironment, and support re-epithelialization [116,118], but more advanced formulations can have improved regenerative effects through tissue remodeling and improved healing quality [117,122,123].

It should also be noted that some of the mentioned studies in Table 4 were conducted during the late 1990s and early 2000s [115,117,118,124]. These studies provided earlier clinical evidence supporting the use of HA-based therapies for burns, diabetic foot ulcers, venous ulcers, and radiation injury and therefore remain important from a historical perspective. However, it should be noted that the formulations evaluated in these earlier studies may have been less sophisticated than those in current studies and that these studies were conducted before the adoption of many current standards for wound healing trials, such as more rigorous reporting practices and standardized clinical endpoints.

Overall, the evidence supports a therapeutic role for HA biomaterials; however, there is still a need for broader studies across multiple centers, using standardized assessment methods and direct comparisons of different HA formulations to identify the most effective treatment strategies.

### 5.3. Clinical Safety Considerations

Despite the biocompatibility of HA-based biomaterials and their low immunogenicity, due to the abundant presence of HA in the ECM, adverse effects can still occur in specific cases, especially with chemical modifications, crosslinked structures, and composite scaffolds. The safety profile of HA-based materials depends on factors such as MW, degree of chemical modification, residual crosslinking agents, sterilization procedures, and the patient’s immune status. Allergic and hypersensitivity reactions are relatively uncommon because native HA is naturally occurring within the body. Despite this, inflammatory responses may occur due to impurities or contaminants introduced during fabrication [125,126].

Although rare, foreign body granulomas can occur after the implantation or injection of HA-based biomaterials. These granulomas exhibit chronic inflammation around the biomaterial, with macrophage activation and multinucleated giant cells. According to recent findings, these granuloma formations may be due to partial degradation, high crosslink density, contamination, or prolonged persistence of HA derivatives within tissues [127]. Therefore, careful consideration is needed during fabrication and rigorous testing to avoid these potential complications.

The interaction between HA biomaterials and microorganisms continues to be studied. Although HA is part of the body’s mechanism for maintaining tissue hydration and aiding wound healing, HA-containing matrices can serve as sites of bacterial colonization when antimicrobial protection is insufficient. Moreover, infections associated with biofilm formation are a problem with HA-based biomaterials, especially when long degradation periods or implants are involved. The risk is higher in immunocompromised patients, including individuals with diabetes mellitus, who often suffer diabetic wounds. In these populations, local infections can progress more rapidly [125,128]. To mitigate these risks, researchers are now incorporating antimicrobial agents into HA-based wound dressings, such as AgNPs, chitosan, or antibiotic-loaded delivery systems [125,129]. This strategy is supported by earlier works on antimicrobial polysaccharide-based hybrid biomaterials [130,131,132]. In addition, other risk mitigation strategies could involve strict aseptic manufacturing, sterilization validation, and patient-specific risk stratification before clinical applications [125,129].

## 6. Current Limitations

Despite extensive research on the positive effects of HA biomaterials on wound healing, several factors limit their full utilization. Many authors discuss these issues in their articles and clinical studies, noting that further standardization of formulations and improvements in trial design are required. While HA is believed to have bacteriostatic properties, the relationship between HA and infection control appears context-dependent. In some instances, HA-based dressings do not provide sufficient protection against bacterial colonization. HA-based dressings may be inefficient when used on chronic wounds with biofilm presence. The bacteriostatic properties of native HA are minimal, while its ability to retain significant moisture levels makes the HA matrix attractive to pathogens [1,133,134]. In a recent study, researchers note that the interaction between HA and microorganisms is dual in nature: HA may inhibit pathogen virulence factors but cannot eradicate pathogens. Therefore, HA must be functionalized with antimicrobial substances [125]. Della Sala et al. also support that HA-based dressings alone are often insufficient in infected wounds and require functionalization to control bacterial load [134].

Despite their numerous advantages, HA-based materials also have some limitations, the main one being rapid degradation. Natural HA is highly susceptible to degradation by hyaluronidase and oxidative stress. This makes HA last only briefly at the wound site, which is not always desired, since the healing process takes longer to complete [59,135]. According to Lin et al., HA’s instability limits its use as a stand-alone biomaterial, so it should be modified or crosslinked for effective applications [133]. In contrast, excessive crosslinking may lead to incomplete degradation of the biomaterial, potentially impairing proper tissue remodeling. As observed by Hussain et al., controlling the degradation rate remains one of the biggest challenges in biomaterial design [136].

In addition to degradation kinetics, the long-term safety of chemically modified HA materials has received limited attention. While crosslinking methods improve mechanical stability and increase retention at the wound site, the metabolic fate of many chemically modified HA derivatives in vivo remains poorly characterized. These materials, during degradation, could produce non-native oligomers and degradation products with biological effects different from those of native HA. Also, the crosslinking agent remaining after synthesis could cause inflammatory reactions. The literature states that the evaluation of the biocompatibility of crosslinked HA biomaterials should be conducted on the biomaterial itself, while also taking into account its degradation products [137,138].

Another important challenge is the synchronization between biomaterial degradation and the phases of wound healing. Ideally, HA-based materials should provide rapid structural support during hemostasis and inflammation, maintain bioactivity throughout the proliferative phase, and gradually disappear during tissue remodeling. In reality, the degradation profiles of most currently available HA-based systems remain constant and cannot adapt to each healing stage. Degradation that is too rapid can cause premature loss of mechanical and biological activity of the material, while degradation that is too slow can affect ECM remodeling and tissue reconstruction [129,139,140]. Therefore, the development of smart HA-based biomaterials with adjustable or stimuli-responsive degradation profiles represents an important research direction.

One of the reasons that some people respond differently to HA-based treatments lies in individual biological differences. Not every person processes the substance at the same rate. This variability arises from factors such as differences in HA molecular weight and formulation; variability in crosslinking density and purity; and patient-specific factors such as age, comorbidities, and wound type [106,141].

Huerta-Ángeles et al. reported that inconsistencies in HA preparation and physicochemical properties can lead to different biological outcomes, and this complicates the interpretation of clinical results [1].

There are also limitations in preclinical and clinical studies, including small sample sizes, short follow-up periods, and a lack of standardized endpoints [106,142,143,144]. Constantin et al. also emphasized that the available evidence from studies is often limited by differences in study designs and short follow-up periods, which hinder direct comparisons of outcomes. It also makes it difficult to assess the long-term effects of HA-based biomaterials. In addition to these, variations in HA’s MW, concentration, crosslinking strategy, and delivery format further complicate comparisons across studies. These limitations show that there is still a need for standardized methodologies and longer follow-up periods [82].

Based on these noted limitations, one of the most significant appears to be rapid degradation and insufficient control of the wound microenvironment. The short residence time of native HA can reduce therapeutic efficacy. This can occur mainly in chronic wounds, which require longer healing times and therefore prolonged treatment periods [59,133]. To address this issue, recent research has focused on chemical modification strategies, including methacrylation, esterification, and advanced crosslinking methods, which improve mechanical stability and allow controlled degradation profiles [54,59]. To address concerns about infection control, researchers have developed systems incorporating antimicrobial agents that enable the biomaterial to combine regenerative and antibacterial functions [53,134].

Achieving consistent clinical outcomes remains challenging due to variability in both biomaterial properties and patient-related factors. To address this, efforts are currently underway to standardize formulation, improve physicochemical characterization, and develop methods that allow tailoring the treatment to each individual, although more research is still necessary [1,22,145,146].

## 7. Emerging Applications and Future Directions

With the discovery of new paths, hydrogel-based biomaterials now go beyond basic wound protection by integrating drug delivery, sensing, and regenerative signaling. Recent research shows an interest in designing intelligent and personalized HA materials [147,148].

### 7.1. Stimuli-Responsive HA Systems

One standout concept involves creating smart hydrogels from HA that change behavior in response to wound conditions, specifically those that react to external triggers. These stimuli-responsive materials are sensitive to changes in pH, temperature, enzymes, and reactive oxygen species (ROS), enabling the release of therapeutic drugs [149]. There are cases in which the wound environment shows inflammation and high levels of ROS. In these cases, the response of the smart hydrogels becomes even more beneficial because it could regulate the hydrogel’s degradation and therapeutic release in real time [149]. Additionally, HA hydrogels have been developed with self-healing properties that can repair themselves following mechanical damage. These types of hydrogels are considered suitable for dynamic tissue conditions, as this process mimics the natural ECM, improves cellular response, and promotes tissue repair [150].

### 7.2. HA-Based Nanotechnology and Targeted Delivery

In the field of nanotechnology, HA can be used to create targeted drug delivery systems. HA NPs can leverage their natural mechanisms, such as HA binding to CD44 receptors, to increase cell penetration and target the desired tissue [151]. There are also recent findings that prove that HA NPs can be very effective in treating chronic wounds by increasing the stability, permeability, and controlled release of drugs [152]. Researchers have developed nanosystems using HA NPs combined with nanofibers and inorganic NPs, which can have multiple functions. They can provide antimicrobial and angiogenic activity while also providing structural support [151].

### 7.3. Gene Delivery and Advanced Therapeutics

Another emerging application for HA-based systems is for gene delivery and molecular therapies. These include siRNA, miRNA, and plasmid DNA. Genes tied to inflammation, blood vessel formation, and ECM remodeling can be adjusted using this approach. Unlike viral vectors, HA-based delivery systems offer several additional benefits, including improved biocompatibility and reduced immunogenicity. One important advantage is that these hydrogels release genetic material directly at the site over an extended timeframe. This supports sustained activity, which is essential for chronic wounds [153,154].

### 7.4. Personalized Regenerative Therapies

Future research may focus on personalized HA therapies. Since every person is different, personalized therapies could suit specific conditions and wound types for each individual. Patient cells can be isolated, cultivated, and incorporated into customized HA scaffolds to produce personalized implants with biological compatibility. For example, autologous cells, such as fibroblasts, keratinocytes, or mesenchymal stem cells, could be cultivated and incorporated into HA matrices. This would create a personalized, biocompatible system that reduces immunogenicity and has regenerative effects [145,149].

Though once limited, methods now evolve through biofabrication and computational design. These new methods focus on 3D bioprinting and artificial intelligence (AI)-assisted scaffold engineering to create patient-specific therapies with HA. This can aid in tailoring HA biomaterials to the geometric shape, depth, and microenvironment of the wound [155]. Other advancements include the field of wound diagnostics and monitoring. These are important in the development of personalized treatment strategies. HA-based wound healing treatments have now been developed to incorporate biosensors. These biosensors can detect wound-site-specific biomarkers, such as pH, temperature, oxygen concentration, and inflammatory molecules, which allow real-time assessment of the healing process [156,157,158,159].

The use of AI and machine learning in this context is becoming more common and accepted because it represents a non-invasive method for predicting healing outcomes. Predictive models can learn from relevant sources and estimate clinical outcomes. This approach also does not require any living models. Using AI, large volumes of data from sources such as clinical imaging and molecular markers can be analyzed to develop HA-based treatments [160,161]. Other significant emerging applications of HA-based systems are presented in Table 5.

## 8. Conclusions

The use of HA as a biomaterial platform for soft tissue repair and wound healing has proven to be very versatile. Besides its biological activity and great physicochemical properties, it exhibits excellent capacity to modify its chemistry, and it also has a good safety profile. Its signaling activities, based on molecular weight, regulate inflammation during the healing process, while interactions with the receptors CD44 and RHAMM initiate vital cellular functions in fibroblasts, keratinocytes, and endothelial cells responsible for regeneration. Today, numerous biomaterials are built on HA, from hydrogels whose properties can be modified to have the desired effects, such as controlled degradation, to composite scaffolds with better stability, which can serve as drug delivery systems.

It is important to note that developments in materials science have led to the creation of chemically modified HA matrices and composites with improved properties compared to simple HA. These can address most of the inherent deficiencies of natural HA, particularly its low mechanical strength and rapid degradation. The addition of biomaterials such as collagen, chitosan, and other polymers enabled the development of biocompatible scaffolds with properties closely resembling those of the native ECM. At the same time, extensive research has led to the development of new HA-based drug delivery systems, which can be in the form of NPs, expanding HA’s applications. These range from tissue engineering to modifying the wound microenvironment. Preclinical and clinical trials have demonstrated the effectiveness of HA-based materials in the treatment of burns, diabetic foot injuries, chronic ulcers, and other chronic wounds. Several commercially available products have already proved their efficacy and safety.

However, despite the numerous benefits of HA, the current evidence base still has some limitations. These include the absence of RCTs with a sufficient sample size and clear outcome criteria. Moreover, issues related to infection control, differences in degradation rates, and patient-to-patient variation emphasize the importance of more controlled and extensive clinical testing.

Moving forward, research is advancing rapidly towards developing HA-based materials that integrate multiple functions, such as drug delivery, biosensing, and regenerative signaling, into a single system. Technologies such as smart hydrogels, nanomaterials, and even computer design and AI are advancing HA material research, leading to novel approaches that can adapt their properties to wound conditions and be personalized for each patient.

## Figures and Tables

**Table 1:** A summary of HA functions in different wound healing phases.

Wound Healing Phase	HA Characteristics	Biological Effects	Ref.
Inflammatory phase	Predominantly LMW-HA fragments	Recruitment of immune cells; stimulation of pro-inflammatory cytokines (IL-1β, TNF-α); activation of CD44 and TLR signaling	[29]
Proliferative phase	High HA concentration; mixed MW distribution	Fibroblast proliferation; collagen synthesis; keratinocyte migration; angiogenesis via VEGF upregulation	[27]
Remodeling phase	Increased HMW-HA; reduced overall HA levels	Resolution of inflammation; ECM remodeling; reduction in fibrosis and scar formation	[36]
All phases	Highly hydrated, viscoelastic matrix	Maintains a moist environment; facilitates cell migration; supports growth factor diffusion	[28]

Abbreviations: HA, hyaluronic acid; LMW-HA, low-molecular-weight hyaluronic acid; HMW-HA, high-molecular-weight hyaluronic acid; IL-1β, interleukin-1 beta; TNF-α, tumor necrosis factor-alpha; CD44, cluster of differentiation 44; TLR, Toll-like receptor; ECM, extracellular matrix; VEGF, vascular endothelial growth factor; MW, molecular weight.

**Table 2:** Examples of composite HA scaffolds and their biological effects.

Composite Type	Experimental Model	Characteristics	Biological Effects	Ref.
HA–collagen scaffold	In vivo rat full-thickness wound model	Improved swelling, moisture retention, and delayed degradation (~50% degradation at 16 days vs. near-complete degradation of CHS at 8 days).	Promotes fibroblast proliferation, angiogenesis, and ECM remodeling. Increased diabetic wound closure ~95% at day 21 for CHS-PDA-2@EGF formulation.	[73]
HA–EGF decellularized scaffolds	In vivo rabbit full-thickness rectangular wounds	HA improved EGF retention and sustained release compared with unmodified scaffolds. ELISA validation showed accurate HA quantification (496 μg/mL measured vs. 500 μg/mL expected).	The HA/EGF-functionalized decellularized scaffold achieved wound healing rates of 49.86%, 70.94%, and 87.41% on postoperative days 10, 15, and 20, respectively, outperforming both the scaffold-only group (29.26%, 42.80%, and 70.14%) and the Vaseline oil gauze control group (19.19%, 38.37%, and 59.10%).	[74]
PLA–HA–valsartan–ascorbic acid scaffold	In vivo alloxan-induced diabetic rats	A porous nanofibrous scaffold (63.90–79.43% porosity), promoting oxygen diffusion, exudate management, and cell attachment. The scaffold exhibited good mechanical properties for wound-dressing applications (tensile strength of 0.63–1.12 MPa) and high thermal stability (up to 329.18 °C).	Highest re-epithelialization rate (85.5% ± 1.7%) compared to controls. Increased wound healing and reduced inflammatory cell infiltration.	[75]
HA–collagen–Propanolol scaffolds	In vitro fibroblast scratch assay; in vivo streptozotocin-induced diabetic rat model	An interconnected porous collagen–HA scaffold (77–133 μm pores) with high swelling capacity (2914%), compressive strength of 35.2 N·mm; demonstrated controlled drug delivery.	92.3% wound closure within 24 h in vitro. In vivo*:* showed 84% closure within 7 days, reaching almost full healing, and 99.7% at day 20. Improved collagen deposition and increased expression of VEGF.	[76]
HA–PRP scaffold	In vivo human chronic ulcers (diabetic and vascular)	The 3D matrix supported fibroblast colonization, ECM deposition, angiogenesis, and tissue remodeling; it maintained a favorable wound microenvironment.	The scaffold showed better re-epithelialization (96.8% ± 1.5%) compared to the control group (78.4% ± 4.4%). Increased granulation tissue formation, improved cosmetic outcomes, and reduced pain.	[77]
Hexamethylene diisocyanate–Pluronic F127 copolymer–HA composite hydrogel loaded with EGF and PHMB	In vitro NIH/3T3 murine fibroblasts	Rapid sol–gel transition (~40 s) at 37 °C. Porous structure (pore size: 5.16 ± 2.03 μm; porosity: 70.5%), sustained integrity (>70% remaining after 7 days), and controlled degradation (27.6% degradation after 7 days). The formulation provided prolonged antimicrobial and growth factor delivery.	The growth rates of NIH/3T3 cells were accelerated by 1.4-, 1.8-, and 1.9-fold. The composite demonstrated antibacterial activity, low toxicity, and a temperature-dependent release profile.	[78]
HA–Chondroitin Sulfate Dynamic Thiol–Aldehyde Addition Hydrogel	In vitro HaCaT, normal human dermal fibroblast (NHDFs) cells	Self-healing capability and on-demand dissolution (<30 min). Increasing the aldehyde content increased crosslinking density, resulting in a 6.5-fold increase in the storage modulus. The hydrogel tolerated strains up to 32–158% and completely restored its structure after mechanical disruption.	Biocompatible and demonstrated increased cell proliferation, showing only a few dead cells after 72 h from hydrogel contact. Rapid gelation (within seconds) and even coverage of irregular wounds.	[79]
PLGA–gelatin–HA scaffolds	In vitro rat cell culture; in vivo rat full-thickness wound model	The crosslinked nanofibrous scaffold with increased fiber diameter (716 ± 14 nm) maintained structural integrity during 7-day culture and exhibited 14.7-fold higher elongation capacity than the PG scaffold.	Adding HA to PLGA–gelatin (PGH) scaffolds increased ASC proliferation 4.0-fold compared to PLGA–gelatin (PG) alone. Relative wound area at day 14 was 9.4 ± 2.1% for PGH/ASCs compared with 15.6 ± 3.0% (PGH), 25.5 ± 3.4% (alginate), and 36.7 ± 3.9% (gauze). Increased angiogenesis, re-epithelialization, collagen deposition, and reduced scar formation.	[80]

Abbreviations: HA, hyaluronic acid; CHS, collagen–hyaluronic acid scaffold; PDA, polydopamine; EGF, epidermal growth factor; ELISA, enzyme-linked immunosorbent assay; PLA, polylactic acid; VEGF, vascular endothelial growth factor; ECM, extracellular matrix; PRP, platelet-rich plasma; PHMB, polyhexamethylene biguanide; NIH/3T3, National Institutes of Health 3T3 mouse fibroblast cell line; HaCaT, immortalized human keratinocyte cell line; NHDFs, normal human dermal fibroblasts; PLGA, poly(lactic-co-glycolic acid); ASCs, adipose-derived stem cells; PG, PLGA–gelatin scaffold; PGH, PLGA–gelatin–hyaluronic acid scaffold.

**Table 3:** Preclinical studies for HA-based biomaterials in wound healing and their findings.

Study Model	HA-Based Material	Key Findings	Ref.
In vitro primary human gingival fibroblasts	HA and polynucleotide (PN) hydrogel	Accelerated cell migration and complete gap closure (within 48 h). Clonogenic efficiency increased from ~5% in controls to ~15% in PN and PN + HA. PN + HA promoted more dense colonies (*p* = 0.0098) and mixed colonies (*p* = 0.02) than controls. PN + HA increased collagen type I and III expression.	[96]
In vitro HaCaT keratinocytes and NHDFs	HA formulations (varied MWs, ranging from 2 to 2290 kDa)	Keratinocyte proliferation increased from 86% to 122% with increasing HA MW. Increased proliferation was associated with HA having an MW above 1000 kDa. HMW-HA (2290 kDa) showed better effects in the proliferation of keratinocytes and fibroblasts and promoted wound closure in 24 and 48 h when compared to MMW-HA and LMW-HA. At days 1 and 3, HMW-HA showed improved wound closure when compared to the controls (*p* < 0.05).	[27]
In vivo diabetic mouse model	Glucose-responsive HA derivative (HAMA-PBA) antioxidant hydrogel	Glucose-responsive HAMA-PBA/catechin (HMPC) reduced intracellular ROS levels. It promoted angiogenesis through increased VEGF and CD31 expression and modulated inflammation by decreasing IL-6 and increasing IL-10 levels. In diabetic wound models, HMPC demonstrated faster wound healing, within 3 weeks, outperforming the other treatment groups.	[100]
In vivo rat full-thickness skin wound model	HA–poloxamer hydrogel	HA–poloxamer hydrogel exhibited sol–gel transition at 30 °C (vs. 37 °C for POL alone), allowing rapid film formation at body temperature. Wound healing was accelerated, with the wound almost completely healed by day 14. The hydrogel promoted fibroblast accumulation, granulation tissue formation, angiogenesis, and collagen deposition.	[102]
In vivo full-thickness wound model	HA–dopamine composite hydrogel	Improved wound healing in HA-DA-CS/Zn-ATV hydrogels, with more than 94% wound closure in 14 days. Angiogenesis and hair follicle regeneration were increased: neovessel and hair follicle numbers were 1.57-fold and 3.05-fold higher than those in the control group.	[103]
In vivo diabetic mouse wound model/in vitro HaCaT keratinocytes, fibroblasts	HA-based delivery platform incorporating conditioned medium (CM) from human foreskin-derived dermal stem/progenitor cells (hFDSPCs)	Improved re-epithelialization, angiogenesis, collagen deposition, collagen maturation, and reduced inflammation. In vitro, hFDSPC-CM improved keratinocyte and fibroblast proliferation and migration, HUVEC tube formation, and promoted ECM regeneration.	[104]
In vivo diabetic mouse models/in vitro HaCaT keratinocyte cells	HA–curcumin conjugate	HA–curcumin increased keratinocyte viability, whereas free curcumin reduced viability by ~45%. Antioxidant activity reached ~38 μmol ascorbic acid, compared with ~29 μmol for free curcumin. In diabetic mice, wound closure after 14 days was ~96% with HA–curcumin, compared with ~81% for HA alone, ~55% for curcumin alone, and ~24% in untreated controls.	[105]

Abbreviations: HA, hyaluronic acid; PN, polynucleotide; HaCaT, immortalized human keratinocyte cell line; NHDFs, normal human dermal fibroblasts; MW, molecular weight; LMW, low MW; MMW, medium MW; HMW, high MW; HAMA, hyaluronic acid methacrylate (methacrylated hyaluronic acid); PBA, phenylboronic acid; HMPC, HAMA–PBA/catechin hydrogel.

**Table 4:** Clinical studies involving HA-based biomaterials and their outcomes.

Wound Type	Product/Formulation	Study Design	Key Findings	Ref.
Second-degree burns	HA–silver sulfadiazine cream (HA-SSD; Connettivina Plus) vs. 1% silver sulfadiazine cream (SSD)	Multicenter, multinational, randomized, double-blind, controlled, parallel-group clinical trial; 111 patients; follow-up for up to 4 weeks.	Both groups were completely healed, except one in the SSD group. Re-epithelialization was faster in the HA-SSD group, with 4.5 days less than the use of SSD alone.	[115]
Partial-thickness facial burns	HA-based dressings	Prospective clinical study, single center; 54 hospitalized burn patients, treated between 2014 and 2017; 6-month follow-up.	Median healing time was 9 days (IQR: 7–12), while median hospital stay was 7 days (IQR: 3–15) overall and 4 days (IQR: 2–7.5) for patients with isolated facial burns. At 6 months, 94% (51/54) of patients achieved a Vancouver Scar Scale score of 0, indicating great cosmetic outcomes. No significant safety concerns were reported.	[116]
Diabetic foot ulcers	HA dressing vs. conventional dressing	Prospective, randomized, placebo-controlled, single center; 34 patients; follow-up: 12 weeks.	HA dressing led to much better results than conventional dressing. In the HA group, 84.6% of ulcers (11 out of 13) completely healed, while only 41.6% (5 out of 12) healed in the control group. Wounds in the HA group also healed faster (*p* = 0.022) and reached 50% size reduction in just 21 days, compared to 39 days in the control group (*p* = 0.0127).	[110]
Diabetic foot ulcers	HYAFF-11 autologous dermal/epidermal grafts	Controlled randomized study; 79 patients; follow-up: 11 weeks.	Complete healing was achieved in 65.3% of the treatment group vs. 49.6% in controls. The mean time to wound closure was shorter with HYAFF-11 grafts (57 vs. 77 days). For dorsal ulcers, healing rates were higher in the treatment group (67% vs. 31%; *p* = 0.049. No adverse events were reported.	[117]
Radiation dermatitis	HA cream vs. placebo	Randomized, double-blind, placebo-controlled clinical trial; 134 patients, prophylactic treatment administered during radiotherapy.	The HA group showed much lower radio-epithelitis scores (*p* < 0.01 during weeks 3–7; *p* < 0.05 at weeks 8 and 10). No reported significant difference in tolerance between groups.	[118]
Split-thickness skin graft donor sites (acute wounds)	HA-based dressing combined with polyurethane foam vs. polyurethane foam dressing alone	Retrospective clinical comparative study; 41 patients; follow-up: 6 months.	HA treatment accelerated wound healing; larger wound closure areas were observed at days 7 and 14 compared with controls (*p* < 0.05). At 1 month, the control reported more crusted wound areas. At 6 months, the HA group reported improved scar appearance compared to the control.	[119]
Palatal (oral surgical) wounds	HA gel vs. injectable platelet-rich fibrin	Randomized clinical trial; 67 patients; follow-up: 3 months.	Comparable efficacy. HA demonstrated better healing outcomes and lower morbidity. Complete epithelialization was improved in both treatment groups (*p* < 0.05). HA provided a simpler and less invasive alternative.	[120]
Experimental gingival surgical wounds	0.8% HA gel vs. no treatment (NT)	Randomized, split-mouth, double-blind clinical trial; 8 patients; gingival biopsies collected 24 h post-surgery.	Median EHS was 9.5 in the HA group vs. 7 in the NT group at 24 h, and 10 vs. 9 at 1 week (*p* < 0.05). HA results indicated increased ECM and collagen maturation during the early healing phase.	[121]
Chronic wounds of mixed etiology	HA + Vibrio alginolyticus collagenase ointment (Bionect Start)	Retrospective observational study; 70 patients; follow-up: 8 weeks.	Debridement efficacy increased from 26% after 2 weeks to 93% after 4 weeks. Complete wound healing was achieved in 62 of 70 patients (88.6%) within 8 weeks. Reduced necrotic tissue, ulcer area, pain, and swelling.	[122]
Chronic vascular leg ulcers	0.05% HA-impregnated gauze pad vs. standard care	Prospective, multicenter, multinational, randomized, double-blind, controlled trial; 168 patients; follow-up: 20 weeks.	The HA-treated group had higher healing rates than the control group (39.8% vs. 18.5%; *p* = 0.002). Complete healing was observed in 33/82 patients in the HA group, compared with 15/86 in the control group.	[109]

Abbreviations: HA, hyaluronic acid; SSD, silver sulfadiazine; IQR, interquartile range; HYAFF-11, benzyl esterified hyaluronic acid derivative.

**Table 5:** Emerging applications of HA-based systems.

Application	Experimental Model	HA-Based System	Findings	Ref.
Nanocomposite systems for electrostimulated wound healing	In vivo excisional wound model in Wistar rats	HA–gold NPs	Accelerated wound closure, increased collagen deposition, and improved angiogenesis in experimental wounds; reduced pro-inflammatory cytokines; increased anti-inflammatory cytokines (IL-4, IL-10) and growth factors; decreased oxidative stress markers.	[162]
Multifunctional hydrogel microneedles with real-time monitoring	In vitro characterization and in vivo diabetic mouse wound model	HA-based microneedle patches	Integrated photothermal antibacterial therapy, exudate management, and real-time pH monitoring. The patch reduced wound pH from 7.4 to 5.9, generated a 23 °C local temperature increase, and achieved 99.9% wound closure in vivo; wound status could be monitored by smartphone.	[163]
miR-223*-loaded HA NP hydrogels	In vitro characterization and in vivo acute excisional wound mouse model	Hydrogel containing HA NPs with miR-223-5p mimic (miR-223*)	Promoted macrophage polarization toward the anti-inflammatory M2 phenotype; accelerated wound healing by promoting vascularized skin formation; reduced inflammation; demonstrated strong tissue adhesion.	[164]
Stimuli-responsive HA hydrogels	In vitro characterization and in vivo diabetic infected rat wound model	HA-based ROS-responsive systems	Controlled degradation and drug delivery: controlled release of calcium ions and tannic acid, providing antioxidant, anti-inflammatory, and antibacterial effects (>99% inhibition of *S. aureus* and *E. coli*); achieved ~95% wound closure by day 14 in vivo compared with ~65% in the control group.	[165]
Wearable wound monitoring systems	In vitro characterization and biological evaluation	PLA-HA crosslinked with Fe^3+^ ions	Hydrogel incorporating 0.6% HA had improved tensile strength, toughness, elasticity, and self-healing; had antibacterial and antioxidant activity; promoted wound healing; functioned as a wearable strain sensor through linear resistance changes under mechanical deformation.	[166]
Fast self-healing hydrogel	In vitro characterization and in vivo full-thickness skin wound model	Double-dynamic HA hydrogel	100% self-healing efficiency within 30 min at physiological temperature, through a dual dynamic network of acylhydrazone, disulfide, hydrogen, and ionic bonds; excellent cytocompatibility; increased granulation tissue formation, collagen deposition, re-epithelialization, and neovascularization.	[167]

Abbreviations: HA, hyaluronic acid; NPs, nanoparticles; miR-223*, microRNA-223 mimic (miR-223-5p); ROS, reactive oxygen species.

## Data Availability

No new data were created or analyzed in this study. Data sharing is not applicable to this article.
